# Adapting Interventions for Early-Onset Dementia: Supporting Frontotemporal Degeneration Caregivers

**DOI:** 10.1093/geroni/igaf122.015

**Published:** 2025-12-31

**Authors:** Lauren Massimo, Esther Kane

## Abstract

Caregiving for a person with dementia is demanding and stressful, particularly for caregivers of persons with frontotemporal degeneration (FTD). FTD is a common cause of early-onset dementia with no known cure. Most patients with FTD remain in the community through the end of their lives, depending on informal caregivers to assist them, yet existing interventions are designed for older adults with Alzheimer’s disease. In this interdisciplinary symposium, we highlight approaches taken to adapt interventions to address the specific needs of FTD caregivers. The first session will describe Living and Learning with Advancing FTD (STELLA-FTD), an intervention that tests the efficacy of the ABC (Activator, Behavior, Consequences) approach to manage behavioral symptoms. The second session describes Primary Progressive Aphasia (PPA) Tele-Savvy, designed to help caregivers address communication in persons with PPA. The third session will describe Virtual Caregiver Coach for You (ViCCY), a motivational health coaching intervention initially designed for caregivers of persons with heart failure. ViCCY utilizes a motivational coaching approach to help caregivers to build individualized resources, with the goal of improving caregiver self-care. The last session will describe the FTD Clinical Research Learning Institute (FTD CRLI), a caregiver engagement initiative that was adapted from Amyotrophic Lateral Sclerosis (ALS). The FTD CRLI, developed in partnership with the Association for Frontotemporal Degeneration, empowers those with lived experience to be advocates in the field of FTD research by educating them on the clinical research process. Together these presentations highlight innovative efforts to tailor interventions for unique challenges faced by FTD caregivers.

